# Editorial: Behavioral and Neurophysiological Approaches to Code-Switching and Language Switching

**DOI:** 10.3389/fpsyg.2021.660695

**Published:** 2021-03-09

**Authors:** Jeanine Treffers-Daller, Esther Ruigendijk, Julia Hofweber

**Affiliations:** ^1^Department of English Language and Applied Linguistics, University of Reading, Reading, United Kingdom; ^2^Department of Dutch, University of Oldenburg, Oldenburg, Germany; ^3^Institute of Education, University College London, London, United Kingdom

**Keywords:** code-switching, language switching, cognitive control, executive functions, task switching, event related potentials

One of the unique characteristics of bilinguals is that they can freely switch between languages, both between and within utterances, a phenomenon that is generally described as code-switching (CS). Since the seminal papers of Pfaff (1979) and Poplack (1980) many linguists working on CS have focused on where switching can take place in a sentence and attempted to formulate (universal) linguistic constraints on this behavior. This branch of research into the linguistic characteristics of CS has led to in-depth insights into the variability in CS patterns found in speech communities across the world, to the development of new CS typologies as well as a renewed understanding of the ways in which sociolinguistic factors interact with these typologies (Poplack, 1988; Muysken, 2013).

Although the term *code-switching* is used in both sociolinguistic and experimental studies, in their overview of research techniques used in code-switching research, Gullberg et al. (2009) make a distinction between *internally generated* CS, for which data are collected using corpus linguistic and sociolinguistic techniques, and language switching (LS), which is *externally induced* in a laboratory situation, where respondents switch languages, e.g., in response to an external cue. Researchers interested in LS generally aim to arrive at a better understanding of the ways in which switches are processed rather than the end product of this process. In this branch of research, experimental methods are used for which the stimulus materials as well as the situation under which respondents respond to stimuli are carefully controlled. We believe that sociolinguistic and experimental approaches are complementary in that each brings vital evidence to our understanding of the ways in which bilinguals switch between languages and the cognitive processes supporting this behavior. A better understanding of CS could therefore be achieved if researchers drew cross-disciplinary conclusions, integrating insights based on both linguistic studies of naturalistic CS and on experimental studies of LS, as in Pablos et al. (2019), who test theory-driven linguistic hypotheses on spontaneous data as well as with EEG methodology. We hope that the current Article Collection will help to further this integration, by bringing together interdisciplinary evidence from different research strands in the field.

In recent years, novel psycholinguistic, as well as neuroscientific methods, such as brain imaging and electrophysiological approaches, have allowed researchers to obtain insights into online processing that cannot be obtained using more traditional offline or behavioral methods which rely on the measurement of the end product of processing or measure reaction times (RTs) needed to complete tasks. Methods from psychology and neuroscience have the potential to revolutionize CS research because they provide a more direct insights into the working of bilingual mind than other methods. They make it possible to observe potential relationships between cognitive processes and language use as well as the neurophysiological correlates of these processes much more directly than had been possible so far, which has led to new insights in these fields [see e.g., Christoffels et al. (2007)].

The development of new models of bilingual speech processing and bilingual visual word recognition (Green and Abutalebi, 2013; Green and Li, 2014; Dijkstra et al., 2019) also led to a renewed interest in CS, for example among researchers interested in Cognitive Control and Executive Functions. Work in this field of research focuses on the attentional control mechanisms that are needed to enable bilinguals to switch between languages. Some studies on the relationship between CS and attentional mechanisms have found that CS practices modulate performance on inhibitory control tasks (Hofweber et al., 2016, 2020), while others have failed to reveal a relationship between CS and attentional processes (Kang and Lust, 2019). Further evidence is therefore needed to study the causes of these inconsistencies.

A new line of enquiry focuses on the neurophysiological correlates of CS with the aim of analyzing brain reactions to CS in real time (Moreno et al., 2002; Ruigendijk et al., 2016; Zeller et al., 2016; Van Hell et al., 2018; Pablos et al., 2019). These studies have the potential to shed more light on the psychological reality of different types of CS, on the magnitude of the processing cost involved in CS, and on the role of variables that may modulate the processing cost of CS, such as speakers' relative proficiency in the two languages, the direction of the switch (i.e., from L1 into L2 or vice versa), and the typological difference between the languages (processing CS in closely related languages vs. in structurally different languages). Specifically, ERP studies can be used to gain insights into the cognitive processes underlying CS.

In this volume four broad topics are addressed: (1) the relationship between CS or LS and cognitive control; (2) linguistic processing of CS and LS; (3) neural and electrophysiological correlates of switching; and (4) linguistic and orthographic analyses of CS and LS. In the remainder of this Editorial we will present each Part in turn.

The focus of the first Part of this Article Collection is on the relationship between CS or LS and cognitive control. In their study among proficient bilingual adults, Barbu et al. found clear evidence for a positive effect of the frequency of reported LS on cognitive flexibility, but not on alertness or response inhibition. In a similar vein, in a study investigating the Adaptive Control Hypothesis (ACH, Green and Abutalebi, 2013), Lai and O'Brien found positive relationships between the frequency of CS and cognitive control performance. Crucially, the Lai and O'Brien study offers partial support for the ACH, but suggests that the three interactional contexts (single, dual, and dense) distinguished by the model should not be seen as a categorical distinction but placed along a continuum. Interestingly from a methodological perspective, the observed effects were stronger when CS was measured using naturalistic conversational data, than when the CS measure was based on self-reports. Hofweber et al. (2016) investigated the effects of experimentally induced language modes and bilinguals' regular CS habits on proactive and reactive control. They also found support for the ACH in that inhibitory performance in the L2-single-language condition was enhanced, possibly because suppressing the L1 requires heightened levels of inhibition. In a highly innovative study taking into account bilinguals' socio-cultural identities, Treffers-Daller et al. explored the relative contribution of informants' CS habits and their multicultural identity styles, that is the strategies individuals use to manage multiple identities, and found that the latter explained most variance in inhibitory control.

For the last two papers in this Part, attention shifts toward the analysis of cognitive control in bilingual children. In the first of these two, Gross and Kaushanskaya tease apart the interaction between cognitive control, language dominance, and language ability. They found an increase in cross-language intrusions among children with lower cognitive control, particularly in the dual-language context, irrespective of children's levels of language ability. The second paper, by Timmermeister et al. focuses on LS and task switching in bilingual children. While the authors found that response times in the LS and nonverbal switching tasks were related, bilingual children did not outperform monolinguals in cognitive control in this study.

In the second Part of the Article Collection, we turn to linguistic processing of CS and LS, for which a range of experimental techniques and behavioral measures are used. In the first contribution, Beatty-Martínez et al. use Green and Abutalebi's (2013) notion of opportunistic planning and suggest that CS can serve as an opportunistic strategy for optimizing task performance, for which they provide evidence on the basis of data from an innovative CS map task. In the next paper, Suurmeijer et al. use another novel technique, namely auditory sentence matching, to study how switch site and switch directionality affect the processing of CS sentences. Contrary to expectations, only effects of the direction of switching but no effects of the switch site were found. The third paper, Kootstra et al. studies the combined effects of interactive alignment (that is alignment between CS behavior of dialogue partners) and lexical triggering (Clyne, 1980) on bilinguals' CS behavior. On the basis of an experimental task which had not yet been used to study these phenomena, they show that lexical triggering is driven by interactive alignment. In the final paper in this Part, Zhang et al. focus on the differences between the cognitive processes underlying language switches and concept switches using a bilingual picture naming task. They found that trials, which involved semantically unrelated items as well as switching between languages led to the longest naming RTs.

In Part three, the focus is on the neural and electrophysiological correlates of switching. These four studies all follow-up on the already mentioned earlier ERP studies that examined the processing of CS (Moreno et al., 2002; Ruigendijk et al., 2016) by zooming in on some relevant factors. Valdés Kroff et al. asked whether semantic and language unexpectancy result in similar processing effects. Their ERP results clearly differ for the effect of semantically unexpected vs. highly expected words, and for CS in Spanish to English switches, with a classical N400 effect for the semantic manipulation and a late positive component (LPC) for the CS, in line with earlier studies. Additionally, these data were related to self-reported experience with CS, which suggested that certain effects are linked to having less experience with CS.

Zeller compared the effect of switching at different positions in a sentence, a preposition or a noun, in German–Russian listeners. He found clear differences between the positions on the relevant ERP components, indicating that the underlying psycholinguistic processes for these two types of CS are indeed not the same. Vaughan-Evans et al. studied adjective-noun order in Welsh–English nominal constructions. They tested predictions of the Matrix Language Framework (MLF, Myers Scotton, 1993) and the Minimalist Program (MP, Cantone and MacSwan, 2009). The ERP data showed different patterns for MLF vs. MP violations. Furthermore, the data suggested that noun insertion is preferred over adjective insertion supporting MLF. Interestingly, the ERP was also modulated by the Matrix Language: when the ML was Welsh, effects were found that were absent when the ML was English. These two studies thus contribute to our theoretical understanding of the rules that governing intra-sentential CS and they do so by examining language combinations that have not received much attention in neurolinguistic approaches to CS so far. The final paper in this Part took a slightly different approach by examining the role of the social situation in which CS takes place by comparing processing in Spanish–English bilinguals in the presence of another bilingual or in the presence of a monolingual speaker of English. Kaan et al. found that relevant ERP effects were smaller in the presence of a bilingual. This indicates that listeners activate their languages in a bilingual social situation and thus CS lead to less processing cost. These results are important for our understanding of language control (see Green and Li, 2014).

The final Part of the Article Collection consists of two papers with in-depth linguistic analyses of CS and two papers which focus on the effects of language-specific letter sequences (i.e., letter sequences that are illegal in one of the two languages) on word recognition. The linguistic analyses start with a paper by Alexiadou, who offers a detailed study of mixed nominal compounds, showing that one of the two contact languages generally provides the underlying structure, i.e., is the matrix language of the compound. The results from a wide range of language pairs are discussed with a view to informing theory building in word formation. The second paper, by Cacoullos, shows how speakers deploy CS strategies, considering prosodic and syntactic variables at switch points of variable equivalence, as is the case, for example, for switches between main and complement clauses where languages have different requirements regarding the use of complementizers. In the third paper, Duñabeitia et al. investigate to what extent bilinguals from different ages use orthotactic cues to recognize to which language a word belongs, on the basis of an innovative language decision task. They found that bilinguals are very good at detecting orthotactic markedness in their L2 even for pseudowords and that this ability increased with age. While their study focused on languages which share the same alphabet but are orthotactically distinct, Chen and Liu focus on trilinguals who use languages that use different scripts. They found no switch costs in a bilingual lexical decision task, nor did they find evidence for effects of the non-task language on lexical processing. Both papers interpret their results in the light of recent models of bilingual visual word recognition (Dijkstra and Van Heuven, 2002; Dijkstra et al., 2019).

The current Article Collection has brought together cutting edge research in the field of CS and LS. The papers illustrate the importance of ensuring experimental work in the field is informed by insights obtained in more naturalistic circumstances, for example by creating experimental stimuli for psycholinguistic and neuroscientific experiments that are representative for the kinds of switching that are found in the real world in a particular language pair. Conversely, as bilingual corpora are generally small and unlikely to provide the necessary evidence about all switches that are possible in a language pair, experimental methods can help drive forward research into constraints on CS (Munarriz-Ibarrola et al., 2018; Treffers-Daller, 2021). As the current volume illustrates, making links between evidence from naturalistic and experimental approaches is not always straightforward, but the combination of insights from different disciplines can lead to the creation of innovative methods, which shed new light on the key problem of how bilinguals manage to keep their languages separate on some occasions while they can switch freely between languages when the situation allows it. We hope the current volume has also contributed to developing models of processing in bilinguals and multilinguals, an endeavor that is urgently needed in the face of the divergent findings in the field.

## Author Contributions

All authors listed have made a substantial, direct and intellectual contribution to the work, and approved it for publication.

## Conflict of Interest

The authors declare that the research was conducted in the absence of any commercial or financial relationships that could be construed as a potential conflict of interest.
